# Open-Label Placebo Treatment for Cancer-Related Fatigue: A Randomized-Controlled Clinical Trial

**DOI:** 10.1038/s41598-018-20993-y

**Published:** 2018-02-09

**Authors:** Teri W. Hoenemeyer, Ted J. Kaptchuk, Tapan S. Mehta, Kevin R. Fontaine

**Affiliations:** 1 Comprehensive Cancer Center, University of Alabama at Birmingham, Birmingham, AL USA; 2 Program of Placebo Studies and the Therapeutic Encounter, Beth Israel Deaconess Medical Center, Harvard Medical School, Boston, MA USA; 3 Department of Health Services Administration, School of Health Professions, University of Alabama at Birmingham, Birmingham, AL USA; 4 Department of Health Behavior, School of Public Health, University of Alabama at Birmingham, Birmingham, AL USA

## Abstract

The purpose of this 21-day assessor blinded, randomized-controlled trial was to compare an open-label placebo (OLP) to treatment as usual (TAU) for cancer survivors with fatigue. This was followed by an exploratory 21-day study in which TAU participants received OLPs while OLP participants in the main study were followed after discontinuing placebos. Cancer survivors (N = 74) who completed cancer treatment 6 months to 10 years prior to enrollment reporting at least moderate fatigue (i.e., ≥4 on a 0–10 scale) were randomized to OLP or TAU. Those randomized to OLP took 2 placebo pills twice a day for 21 days. Compared to those randomized to TAU, OLP participants reported a 29% improvement in fatigue severity (average difference in the mean change scores (MD) 12.47, 95% CI 3.32, 21.61; P = 0.008), medium effect (d = 0.63), and a 39% improvement in fatigue-disrupted quality of life (MD = 11.76, 95% CI 4.65, 18.86; P = 0.002), a large effect (d = 0.76). TAU participants who elected to try OLP for 21-days after the main study reported reductions in fatigue of a similar magnitude for fatigue severity and fatigue-disrupted quality of life (23% and 35%, respectively). OLP may reduce fatigue symptom severity and fatigue-related quality of life disruption in cancer survivors.

## Introduction

Nearly 14 million cancer survivors report cancer-related fatigue (CRF), an intractable condition with only marginally effective treatments due to its multifactorial and complex nature and poorly-defined pathophysiology^1^. CRF can continue years after treatments end, affecting quality of life and the ability to carry out normal, daily activities^1–5^. Participants in randomized controlled trials (RCT) for CRF comparing active medication to placebo controls often show high placebo responses that are indistinguishable from active component^6–8^. However, it is considered unethical to use placebos in clinical practice because eliciting positive responses is thought to require deception or concealment^9–11^. Furthermore, it seems intuitive that if participants know they are taking placebos, it would not produce benefits.

Recently, five non-deceptive open-label placebo (OLP) studies (i.e., where participants were told they were receiving placebo pills) demonstrated significant improvement for patients with Irritable Bowel Syndrome (IBS), episodic acute migraine attacks, chronic low back pain, allergic rhinitis and depression^12–16^. Although there are some methodological limitations, a recent meta-analysis found overall moderate effect sizes for open-label placebos^17^. While these studies indicate beneficial effects on patient-reported outcomes (PROs), it is unknown whether OLP might reduce CRF. Therefore, we evaluated the effects of OLP for CRF.

## Methods

### Study Design

From August 2015–May 2017, we conducted a 21-day, single site, two-parallel arm RCT to compare the effects of OLP to treatment as usual (TAU) among cancer survivors reporting at least moderate CRF. In addition, as a retention device and secondary exploratory study, we offered the participants randomized to TAU the opportunity to take OLP for 21 days at the conclusion of the main study, while participants originally randomized to OLP group in main study were offered an opportunity to be followed for an additional 21 days to investigate whether any improvement persists after placebo was discontinued. Participants were compensated up to $75 for their time.

### Site, Ethics Statement and Trials Registration

This study was conducted in the University of Alabama at Birmingham (UAB) Comprehensive Cancer Center. The Clinical Trials Review Committee (CTRC) of the Comprehensive Cancer Center and UAB’s Institutional Review Board (IRB) approved this study, and written informed consent was obtained from participants prior to enrollment. The design and procedures of the study were carried out in accordance with the principles of the Declaration of Helsinki. This study was registered with Clinicaltrials.gov on 13/08/15 (NCT02522988).

### Study Population

Patients were eligible if they were older than 19 years old, had a cancer diagnosis, had completed cancer treatment between 6 months and 10 years prior to enrollment, and reported at least moderate fatigue (i.e., ≥4 on a 0–10 scale). Clinically significant CRF is defined as ≥3 with categories for CRF as 0 = no fatigue, 1–3 = mild, 4–6 = moderate, 7–10 = severe fatigue^18,19^. Participants agreed not to change their medications or dosages or to make any major lifestyle changes (i.e., diet or physical activity) during the trial. Participants with uncontrolled medical conditions, such as cardio-vascular disease, hypertension, anemia and diabetes were excluded.

### Recruitment and Enrollment

We identified potential participants through medical record examinations and print and media advertisements. Prospective participants identified through medical records received a letter describing the study.

A research specialist screened potential participants, by phone, for eligibility using an *a priori* script. Potential participants were assessed on a 0–10 scale (i.e., 0 = no fatigue; 10 = as severe as it can be). If the individual’s meet criteria including a fatigue score of ≥4, he or she received more information about the study and, if willing to participate, was scheduled for 2 clinic visits.

### Study Measures and Primary Outcomes

Demographic information was collected during the initial screening telephone call (e.g., age, race, gender, cancer type and stage, time since last treatment).

To assess the effects of OLP on CRF, we used two reliable and well-validated questionnaires for CRF: the Fatigue Symptom Inventory (FSI-14) (α = 0.84–0.96) which measures global fatigue symptom severity and the Multidimensional Fatigue Symptom Inventory Short Form (MFSI-SF30) (α = 0.84–0.93) which measures the extent to which fatigue disrupts quality of life^20,21^. The FSI is scored to provide a global score, with lower scores indicative of lower fatigue. MFSI-SF30 produces 5 domain scores: general, physical, emotional, mental health and vigor, as well as a total score. For simplicity and to preserve power, we report only the total score. Lower MFSI-SF30 scores are indicative of lower level of fatigue-disrupted quality of life. Questionnaires were completed at baseline, Day 21, and during the extension exploratory period (i.e., on Days 28 and 49). Patients were asked if there were any placebo-related side-effects at day at Day 11, Day 21 (main study), Day 39 and Day 49 (exploratory period).

### Power and Sample Size Calculation

Because there have been no other OLP studies that have investigated the use of an OLP for cancer survivors suffering from CRF, an effect size estimate for our primary outcomes is not known. Therefore, we averaged the effect sizes found in an OLP study of IBS (d = 0.78) and, a feasibility OLP study in major depression (d = 0.54) to determine an estimate of d = 0.66^12,16^. Assuming a two-group, two-tailed t-test, type 1 error rate of 0.05 and 80% power, we estimated sample size of 40 to detect an effect size of 0.64. For each outcome, our planned sample size calculations indicated that we would have power of 80% assuming a two-tailed two-sample t-test on the outcomes at day 21, Type 1 error rate of 0.05, and we would need a sample size of 80 to detect an effect size of 0.64. Techniques such as ANCOVA or two–sample independent t-test comparing change scores would be powerful or require less sample size to detect the same effect size depending on the correlation between baseline and follow-up measures^22^. Since the goal of this proof-of-concept study was also to estimate an effect size, our sample size considerations were based on the recommended sample sizes of at least 70 when estimating the pooled standard deviation of the continuous outcome^22^.

### Randomization and Blinding

Before any participant visits, a research specialist, otherwise uninvolved in the study, placed white sheets of paper with 40 “Group 1” (OLP) and 40 “Group 2” (TAU) assignments into 80 opaque envelopes. The envelopes were shuffled and randomly placed in a pre-enrollment allotment of files assigned consecutive numbers. As each qualified participant agreed to enroll in the study, he or she was assigned a consecutively numbered file containing the concealed group assignment. During the first clinic visit, after each participant was consented and received the placebo orientation, (see below) the envelope containing the group assignment was then opened and revealed. Until the envelopes were open at the last moment, the interaction with participants was identical. All assessments were performed by a research assistant blinded to randomized allocation.

### Study Procedures: Placebo Orientation and Intervention

Upon arrival for the first clinic visit (Day 1), each participant completed the informed consent process and study questionnaires. Afterwards, all participants met with the Principal Investigator (PI) (TWH), an oncology health behavior specialist, who delivered a rationale with 4 main discussion points, established *a priori*. The 4 points were: 1) placebo effects are powerful in double-blinded clinical trials and there is some evidence that placebos work even when patients know it’s placebos but we don’t know if they work when honestly prescribed for CRF; (2) placebo responses may be attributed to conditioning, expectancy and biological (e.g., neurological, genetic) factors; 3) an open mind is helpful but unrelated to outcomes that may happen automatically; and, 4) taking the placebo pills as prescribed for 21 days is important (OLP group). The PI delivered these discussion points in a way that fostered emotional support, hope and trust. We also tried to dispel widespread beliefs that placebo effects are negative or unauthentic. Nonetheless, when patients expressed their skepticism about whether placebo pills do anything, which was fairly common, we comfortably acknowledge disbelief is understandable and encouraged them to “see what happens.”

After both groups received the same introduction (which controlled for patient-provider interaction), the PI then opened an opaque envelope revealing the participant’s randomized assignment (i.e., OLP or TAU). If the participant was randomized to OLP, the PI provided pre-packaged pills, clearly labeled “placebos”, along with oral and written instructions to take 2 placebo pills twice per day and how to use the medication diary to track adherence. All participants understood that the placebo pill only contained microcrystalline cellulose and not active ingredients. This was explained during the screening call, during the placebo orientation discussion and when prescribed. Finally, this information was communicated in the written instructions provided to each OLP participant. OLP participants were asked to take 2 placebo pills in the presence of the PI to ensure that there were no swallowing difficulties and advised not to change any other medications. Participants randomized to TAU were asked to continue with their current treatments and were reminded that they could receive the placebo intervention at Day 28 of the study. All participants were contacted by telephone on Day 11 by the PI to inquire about changes in physical condition or medication status and whether participants had additional questions or concerns.

At the main study’s endpoint, the second visit (Day 21), participants met briefly with the research specialist, who was blinded to assignment, and completed the study questionnaires. If participants chose to participate in the extension (exploratory phase) of the study, additional clinic visits were set for the following week (Day 28) and 21 days thereafter (Day 49).

On Day 28, as a reminder, participants who were initially randomized to TAU received identical instructions from the PI as delivered to all participants on Day 1. This meant that this TAU group received the rationale twice. Participants who were randomized to OLP during the main study were scheduled to return for another visit 21 days after the placebos were discontinued. All participants were contacted by telephone on Day 39 by the PI to inquire about changes in physical condition or medication status and to address questions or concerns.

At the final visit of the exploratory study, the blinded research assistant assessed patients (Day 49). Following this, the PI met briefly with the participants to gather anecdotal/qualitative information related to responses to the intervention. (This information will be reported elsewhere.) Finally, a brief educational discussion was held with the participants about additional strategies (i.e., diet, exercise, stress management, sleep hygiene) to manage CRF.

### Statistical Analysis

We used a two-sided two sample t-test assuming unequal variances as primary analysis to test differences between the change scores in the OLP and TAU groups on all outcomes. Statistical significance was tested at the 0.05 level. Since both outcomes were pre-specified we did not account for multiple testing. Changes in FSI and MFSI-SF30 scores during the 3-week exploratory follow-up period (day 28 through day 49) among participants were analyzed using a paired sample t-test for both OLP and TAU arms. For each analysis, we calculated Cohen’s *d* as an estimate of effect size. Given that there was only one drop out (randomized to OLP), missing data was handled using list-wise deletion, which is a valid approach under missing completely at random. Additional sensitivity analyses were done using the non-parametric Wilcoxon rank-sum test. Originally, the study protocol called for an ANCOVA. This analysis was performed for the data used for the main study period and there were no differences found between the ANCOVA and t-test results. For ease of interpretation, we chose to report the t-test results and the ANCOVA outcomes are available upon request.

The datasets generated during and/or analyzed during the current study are available from the corresponding author on reasonable request.

### CONSORT 2010 Guidelines

**Figure 1:**
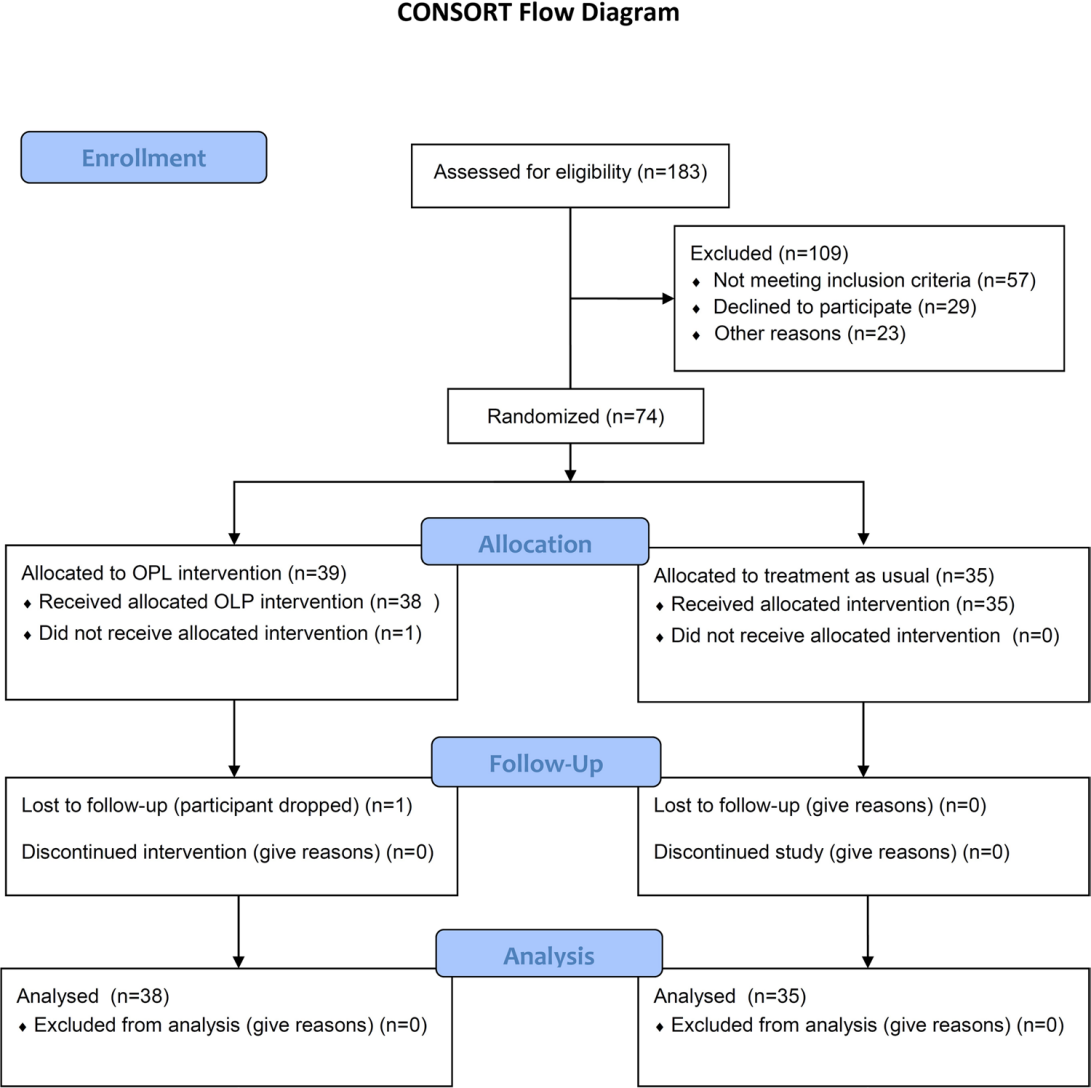
CONSORT Diagram^29^.

This clinical trial conforms to the CONSORT 2010 Guidelines. A completed CONSORT diagram and check list are listed in the manuscript as Fig. 1.

This study is registered withClinicaltrials.gov on 13/08/15 (NCT02522988). https://clinicaltrials.gov/ct2/show/NCT02522988.

## Results

Of the 4,949 medical records reviewed, we identified 544 qualified candidates who met the basic study criteria (i.e., diagnosis, stage, co-morbid conditions) and sent them study announcement letters. Study assistants followed up with phone calls and were able to screen 183 of whom 74 were enrolled. (Our original enrollment goal was 80 but, due to funding limitations, enrollment was stopped at 74). The CONSORT Flow Diagram is shown in Fig. 1.

Of the 74 participants enrolled, 51 (69%) were female with 55 (74%) self-identifying as White, 18 (24%) as African Americans and 1 (1%) as Hispanic. Twenty-eight (38%) reported a moderate level (i.e., 4–6) of fatigue at baseline while 46 (62%) reported a severe level (7–10) of fatigue. The mean fatigue scores reported at screening by OLP and TAU participants was 6.9 and 6.8, respectively. The average length of time since last cancer treatment was 27 months. Table 1 shows the demographic and cancer-related characteristics of the study participants by randomization.

**Table 1:** Baseline Characteristics of Study Participants (N = 73).

Characteristic	Open-Label Placebo (OLP)	Treatment as Usual (TAU)
	(N = 38)	(N = 35)
Age, Mean (SD)	58.4 (11.2)	56 (12.4)
Female, n (%)	28 (72%)	23 (66%)
Race, n (%)		
White	30 (77%)	25 (71%)
African-American	9 (23%)	9 (26%)
Hispanic	0	1 (1%)
Cancer Type, n (%)		
Colorectal/GI	8 (21%)	9 (26%)
Breast	14 (36%)	11 (31%)
Gynecologic	6 (15%)	5 (14%)
Brain	1 (2%)	3 (9%)
Leukemia/Lymphoma	3 (9%)	3 (9%)
Head/Neck	6 (15%)	4 (11%)
Melanoma	1 (2%)	0
Cancer Stage, n (%)		
2	13 (33%)	13 (37%)
3	16 (41%)	10 (29%)
4	6 (15%)	8 (23%)
Un-staged	4 (11%)	4 (11%)
Time since last treatment, months, n (%)		
6–12	4 (10%)	4 (11%)
12–24	9 (23%)	6 (17%)
24–36	4 (10%)	6 (17%)
>36	22 (56%)	19 (54%)
Fatigue rating (0-10) at screening, M (SD)	6.9 (1.6)	6.8 (1.4)

### Change in Outcomes Between Baseline and 21 Days

As shown in Table 2, the average difference in the mean change scores between OLP and TAU for FSI was 12.47 (95% CI, 3.32, 21.61, p = 0.008, Cohen’s *d* = 0.63) and statistically significant. This effect size can be interpreted as 73.6% of the OLP group had a change score above the mean change score of the TAU. We defined a clinically meaningful change for CRF as one with an effect size (i.e., Cohen’s *d*) greater than 0.5 relative to baseline at 3 weeks as defined by Yun *et al*., for CRF^23^. In terms of common language effect size, there is a 67.2% probability that a person picked at random from the OLP group will have a higher (better/improved) change score compared to a person picked at random from the TAU group^24,25^. Participants randomized to OLP also reported a statistically significant difference in the mean change scores in MFSI-SF-30 (i.e., fatigue-disrupted quality of life) in OLP group compared to TAU (MD 11.76; 95%, CI: 4.66–18.86, p = 0.002, *d* = 0.76) (see Table 2). This effect size can be interpreted as 77.6% of the OLP change scores will be above the mean change score of the TAU. In terms of common language effect size, there is 74.5% probability that a person picked at random from the OLP group will have improved change score than a person picked at random from the TAU group. Our findings were robust to non-parametric Wilcoxon rank sum test comparing change scores between OLP and TAU.

**Table 2:** Effects of OLP on Fatigue Symptom Severity (FSI) Scores and Multidimensional Fatigue Symptom Inventory Short Form (MFSI-SF-30) Scores.

Outcome	OLP (N = 38)	Tau (N = 35)	Mean Difference in Change Scores (95% CI)	Cohen’s *d*	P
FSI	Mean (SD)	Mean (SD)			
Baseline	64.3 (23.3)	59.0 (21.1)			
Day 21	45.7 (22.7)	52.9 (24.1)	12.47 (3.32–21.62)	0.63	0.008
**MFSI-SF-30**	**Mean (SD)**	**Mean (SD)**	**Mean Difference in Change Scores (95% CI)**	**Cohen’s** ***d***	**P**
Baseline	33.9 (17.8)	27.4 (19.3)			
Day 21	20.8 (19.5)	26.0 (21.5)	11.76 (4.65–18.86)	0.76	0.002

### Outcomes of Exploratory Follow-Up Phase (Day 28 to Day 49)

Thirty-four TAU participants chose to participate in the exploratory phase, taking OLPs for 21-days from Day 28–Day 49 (see Table 3). The results indicated a significant improvement in change scores with medium effects in FSI (MD = 11.83, SD = 18.11, p = 0.001, *d* = 0.49), as well as in MFSI-SF-30 scores (MD = 7.65, SD = 13.29, p = 0.002, d = 0.36). Surprisingly, among the 37 participants originally randomized to OLP who agreed to be followed for an additional 21 days (i.e., Day 28 – Day 49) after discontinuing the placebos, we found no significant differences in change scores in FSI (p = 0.619) or MFSI-SF-30 (p = 0.733) compared to their post-intervention (Day 21) change scores. Overall, after three weeks participants did not experience a degradation of their improvement.

**Table 3:** Results of Exploratory 21-Day Follow-Up in TAU Participants on Placebos and OLP Participants off Placebos.

Outcome	TAU on Placebo (N = 34)	OLP off Placebo (N = 37)
FSI	Mean (SD)	Mean (SD)
Day 28	50.65 (21.96)	40.40 (27.57)
Day 49	38.82 (25.69)	41.97 (32.41)
**Cohen’s d**	0.49	0.05
**P-Value**	0.001	0.619
**MFSI-SF-30**	**Mean (SD)**	**Mean (SD)**
Day 28	21.53 (20.33)	17.86 (21.05)
Day 49	13.88 (22.12)	18.57 (21.56)
**Cohen’s d**	0.36	0.03
**P-Value**	0.002	0.733

## Discussion

**Figure 2:**
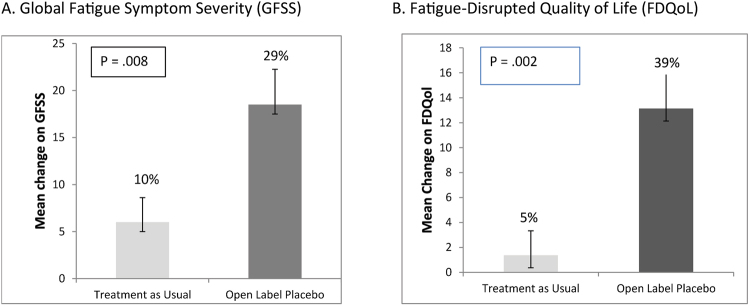
Outcomes by treatment group at 21-day endpoint. (**A**) Mean change scores on global fatigue symptom severity (GFSS). (**B**) Mean change scores on fatigue-disrupted quality of life (FDQoL). Error bars represent standard errors of the mean.

At the end of the main 21-day study, the OLP group reported statistically significant improvements in fatigue compared to the TAU group, suggesting that an OLP treatment may be an effective treatment for CRF. Current pharmacological treatments for CRF are marginally effective or are not significantly better than placebo comparators and, generally, have considerable side effects^3–5^. By comparison, our results indicate participants taking an OLP experienced a 29% improvement in fatigue severity (i.e., FSI) while fatigue-disruption on quality of life (i.e., MFSI-SF30) improved by 39%, with medium and large effect sizes, respectively (see Fig. 2). The average effect size across the two outcomes was 0.7, which translates to mean that 76% of the OLP group will be above the mean of the TAU group and there is a 69% chance that a person picked at random from the treatment group will have a higher change score than a person picked at random from the TAU group. Importantly, these results were clinically meaningful as defined in the literature^23^. Moreover, there were no reported adverse events or side effects.

**Figure 3:**
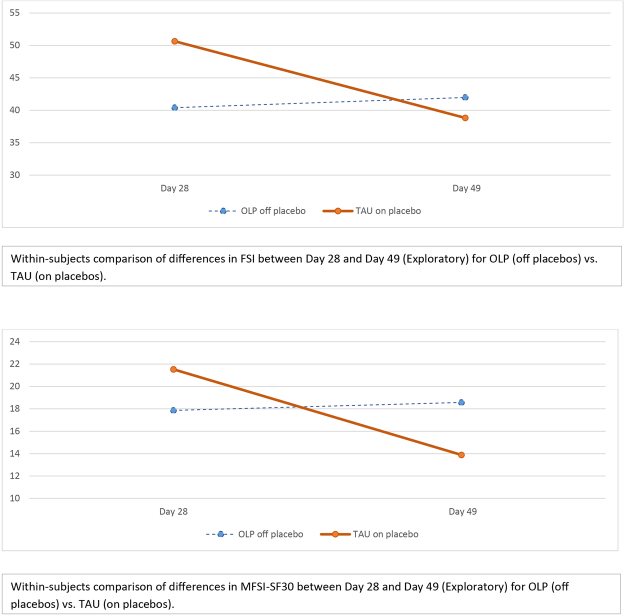
Exploratory Results for Fatigue Severity Inventory (FSI).

Originally, we considered a formal crossover design. We rejected this option for two reasons. First, it was unclear whether there would be a carryover effect with OLP and, if so, how long it would last (the fact that we found a carryover effect for those randomized to OLP confirms that a crossover design would have been inappropriate). Second, we thought it might be necessary to fully discuss the OLP rationale a second time with the TAU group after the 21-day main study which might have a secondary “booster” effect compared to those originally randomized to OLP. Therefore, we decided to conduct a 21-day exploratory crossover extension, as described above, which began one week after the primary study ended. Our exploratory study, in which patients who were randomized to TAU were switched to OLP on Day 28, supports our main study results, in that original TAU group showed a similar magnitude of improvement as those randomized to OLP in our main study in both FSI and MFSI-SF30, 23% and 35%, respectively. With regard to those originally randomized to OLP, at day 48 (28 days after OLP ceased), there was no significant change in fatigue scores compared from Day 21, suggesting that the effects of OLP may be sustained for some time even after placebos are discontinued (see Fig. 3). Interestingly, a member of our team (TJK) has been clinically and unsystematically following individual IBS patients from the previous OLP study^12^ whose symptoms relapsed after discontinuation of OLP and when put back on OLP again remained symptom free for over 12 months of pill taking. While this preliminary finding is exciting, additional work is needed to evaluate the durability of OLP’s effects, as well as to develop and test whether its benefits can be maintained over the intermediate to long-term.

Several issues deserve mention related to OLP. A recent bioethical analysis found that OLP accompanied by informed consent and transparency, unlike deceptive administration of placebo, conforms to normative ethical standards^26^. Patient acceptance also does not seem to be problem. A recent large survey of a cross-section of patients (n = 853) performed at a major US hospital found that nearly 85% considered OLP acceptable if given with honesty by a physician^27^. A smaller focus group study (n = 58) in the UK had similar findings^28^. To our knowledge, there are no data concerning whether physicians would accept OLP if the scientific evidence was robust and compelling.

## Limitations

This proof-of-concept study is subject to a number of limitations, including the relatively small sample size and short duration, which limit inferences that can be made concerning both OLP’s effects and generalizability. Furthermore, given the absence of blinding in the study (except for the assessor) we cannot rule out performance bias. In other words, given the inability to blind participants to study condition, we cannot rule out that this awareness may have influenced their perception and/or detection of a treatment effect. This said, there is no other way to evaluate an un-blinded placebo treatment. Furthermore, the consistency and similar robust findings our study had compared with previous studies in IBS, episodic migraine, and chronic low back pain suggest genuine improvement. However, while our results are positive, OLP’s effects need to be confirmed by larger and more rigorous trials of longer duration among different conditions to explore the extent to which biological, social and behavioral factors might influence responses to open-label placebos.

## Conclusion

Although our results suggest that OLP may be a beneficial treatment for CRF, replication studies are needed, as well as studies exploring how OLP works, why and under what circumstances. Furthermore, efforts should be undertaken to learn whether OLP’s can be successfully applied to a range of PROs in cancer and other populations.

## Electronic supplementary material

CONSORT CHECKLIST

IRB APPROVED STUDY PROTOCOL
