# Supplementary material for: Open-Label Placebo Treatment for Cancer-Related Fatigue: A Randomized-Controlled Clinical Trial
Source: Sci Rep. 2018 Feb 9;8:2784. doi: 10.1038/s41598-018-20993-y (PMC5807541; doi:10.1038/s41598-018-20993-y)
Supplement: Supplementary file 2 — IRB APPROVED STUDY PROTOCOL [file 41598_2018_20993_MOESM2_ESM.doc]

**Comprehensive Cancer Center**

**University of Alabama at Birmingham**

**Birmingham, Alabama**

**Study/Protocol Title:** The Use of an Open-Label Placebo Intervention to Treat Cancer-Related Fatigue in Cancer Survivors

Theresa W. Hoenemeyer, MS, PhD

WTI 220A

Comprehensive Cancer Center

(205) 934-5772

Tgw318@uab.edu

**Principal Investigator:** Theresa Hoenemeyer, PhD

**Biostatistician:** Tapan Mehta, PhD

**Other Co-Investigators**: Kevin Fontaine, PhD

Connie Kohler, DPH

Robin Lanzi, PhD

Julie Locher, PhD

Tapan Mehta, PhD

Carolina Salvador, MD

Ted Kaptchuk, OMD, Consultant

**TABLE OF CONTENTS**

**1.0 Background and Significance 3**

**2.0 Study Objectives 4**

**3.0 Investigational Plan and Procedures 4**

**3.1 Study Design and Population 4**

**3.2 Participants and Recruitment 5**

**3.3 Informed Consent 6**

**3.4 Inclusion, Exclusion, and Withdrawal Criteria 7**

**3.5 Randomization and Interventions (if applicable) 7**

**3.6 Measures 12**

**3.6.1 Primary Study Aim Outcomes 12**

**3.6.2 Secondary Study Aim #1 Outcomes 13**

**3.6.3 Supplemental Aim Outcomes 13**

**3.6.4 Primary Endpoint 14**

**3.6.5 Covariates 14**

**3.7 Statistical Methods 14**

**4.0 Data and Safety Monitoring 16**

**4.1 Ethical Considerations 17**

**4.2 Confidentiality 17**

**5.0 References 18**

**1.0 Background and Significance**

Public Health Burden: Cancer-Related Fatigue

Cancer-related fatigue (CRF) is one of the most distressing symptoms that a person living with a cancer diagnosis experiences[[1]](#endnote-2). The National Cancer Institute’s PDQ ® on CRF states that it is “the most common side effect of cancer treatment with chemotherapy, radiation therapy, or selected biologic response modifiers”[[2]](#endnote-3) . Many studies report the prevalence rate of CRF to be between 14% to 99% for survivors undergoing treatment and 19% to 82% for survivors post-treatment[[3]](#endnote-4), [[4]](#endnote-5), [[5]](#endnote-6), [[6]](#endnote-7), [[7]](#endnote-8), [[8]](#endnote-9). It is pervasive and affects a survivor’s quality of life, social relationships and daily activities. It also places significant hardships on the survivor by limiting how much he or she can work or gain access to the social networks, resources and care that are critical for living with a cancer diagnosis[[9]](#endnote-10). Factors that may be associated with CRF include cancer treatment, anemia, inflammation, medications, sleep disorders, nutrition and psychological distress. Studies have suggested that pharmacologic interventions, exercise, cognitive behavior therapy and education may be treatment strategies. However, none of these has proven to be significantly effective for relieving CRF symptoms.

Open-Label Placebo (OLP) Intervention

Although randomized placebo-controlled drug trials sometime show that placebos produce patient-reported outcomes that are indistinguishable from those observed with active compounds, it is considered unethical to use placebos in clinical practices because eliciting positive responses with placebos is thought to require deception (i.e., treating with biologically inactive compounds without consent) or concealment (e.g. in randomized trials).

However, three recent non-deceptive (open-label) placebo studies –where patients were told they were receiving placebo pills – demonstrated improvement in symptoms for patients with Irritable Bowel Syndrome (IBS) (N=80, Cohen’s d=.79), in depression scores for adults with Major Depressive Disorder (MDD) (N= 20, Cohen’s d=.54) and in pain severity for migraine sufferers[[10]](#endnote-11),[[11]](#endnote-12),[[12]](#endnote-13). While these studies indicate that open-label placebos (OLPs) can influence patient-reported outcomes, it is unknown whether the same holds true for symptoms of CRF for which there are no significantly efficacious treatments. In addition, it is suggested that a certain genetic variation of COMT Val158Met (met/met) correlates with dopamine metabolism and is a potential biomarker suitable for identifying placebo responders[[13]](#endnote-14). (Dopamine metabolism disorder is one of the biochemical mechanisms hypothesized to be a causal factor of cancer-related fatigue[[14]](#endnote-15)).

Significance of this Study: There are serious ethical challenges to using placebos in clinical practice because use involves deception or concealment. A non-deceptive or “open-label” placebo is a sound, safe strategy that harnesses placebo effects for clinical practice in a way that is ethical, transparent and with informed consent. This study is significant because:

(1) It offers a strategy to harness placebo effects in conditions that are prevalent but where there are no effective treatments. If placebo effects can be elicited without deception or concealment, a significant challenge will be removed for the ethical harness of placebo effects in clinical practice.

(2) It is an innovative application of a clinically important but newly developed non-deceptive placebo methodology.

(3) It is a potential treatment for CRF that currently has no efficacious treatment.

(4) It has access to UAB resources, support and expertise.

(5) It addresses an important and ongoing clinical problem that compromises optimal and safe care.

(6) It will provide information on feasibility, effect sizes and factors that contribute to the variability in placebo responses for cancer survivors with moderate to severe symptoms of CRF.

(7) If will provide information that can lead to a larger trial with a substantially longer follow up with a broader array of outcome measures (e.g., cost-effectiveness, potential biomarkers).

(8) It may lead to additional clinical intervention studies for cancer survivors with the potential of enhancing the effectiveness of existing treatments and/or reduce the burden of side effects (e.g., nausea, depression, and neuropathy).

**2.0 OBJECTIVES**

The purpose of this randomized-controlled, crossover pilot trial is to evaluate the feasibility, acceptability and effects of a non-deceptive (open-label) administration of placebo pills for treating CRF. If significant effects are found, we will later determine if the presence of a COMT Val18Met genotype variant predicts placebo responses.

**Primary Objective**

**Specific Aim 1**: Assess the feasibility of an Open-Label Placebo (OLP) for treating CRF in cancer survivors (CSs).

Hypothesis 1: An OLP will be perceived by the majority of CSs screened as credible and acceptable and we will meet our enrollment, retention and adherence targets.

**Secondary Objectives:**

**Specific Aim 2:** Evaluate whether an OLP reduces CRF severity and improves quality of life (QoL) in CSs compared to Observational Controls (OCs).

Hypotheses 2: Over the course of 49 days, CSs receiving the OLP will report significant declines in CRF severity and significant improvement in QoL scores compared to OCs.

**Supplementary Aim**: We will collect and store saliva samples so, should significant OLP effects be obtained, we can evaluate whether a potential biomarker (COMT Val158Met variant) associates with placebo responsiveness.

Hypothesis: The presence of the COMT Val158Met variations (met/met and val/val alleles) will correlate with the placebo responsiveness.

**3.0 INVESTIGATIONAL PLAN AND PROCEDURES**

**3.1 Study Design and Population**

We propose to conduct a pilot randomized-controlled, crossover trial to evaluate the feasibility, acceptability and effect of the open-label administration of placebo pills on CRF and associated psychosocial factors (e.g., emotional health, social health, quality of life, etc.) for cancer survivors (CSs) who have completed all cancer treatments yet continue to experience CRF. Additionally, in an exploratory manner, we propose to collect DNA saliva specimens that we intend to analyze in the future to identify potential biomarkers for predicting placebo responses.

To do this, we will use a 7-week, single site, two-parallel arm, randomized controlled crossover pilot study to determine the feasibility, acceptability and effects of an OLP on CSs who completed cancer treatments at least 6 months prior to enrollment in the study and report a fatigue score of 4 or greater on a 0-10 scale.

We will enroll 80 eligible CS participants who will be randomized into two groups of 40 (Group_1_ and Group_2_). In this crossover study, participants in Group_1_, will receive an OLP (placebo pill) for 21-days and Group_2_ (Observational Controls) will not. After a 1-week washout period, Group_2_ will receive the OLP for 21-days and Group_1_ (Observational Controls) will not (see Figure 1a: Study Design Schema).

**3.2 Participants and Recruitment**

**Participants**: We intend to screen and recruit a sufficient number of individuals in order to enroll 80 cancer survivors, over the age of 19 who received oncology treatments at UAB and who meet our inclusion/exclusion criteria.

**Recruitment:** The primary method for identifying potential participants for this study will be through medical records examination. We will attempt to get a listing of people who were treated for cancer by cancer stage between 2009 – 2016 from the UAB Tumor Registry, the Center for Clinical and Translational Science and IMPACT. We will then compare this record to concurring physician’s patient database and to medical records information contained in IMPACT to identify recruits who meet the inclusion and exclusion criteria.

In order to meet this goal, we will also solicit recruits who received treatment at UAB through the use of flyers, posters and advertisement in UAB newspapers, presentations to local support groups and organizations.

Once we identify a list of prospective participants, we will review the listing with the treating physician. Once the treating physician concurs, we will send the prospective participant a letter signed by both the physician and the PI to offer the opportunity of participating in a study that will test a new, natural, non-pharmaceutical treatment for relieving CRF symptom severity. The letter will contain an opt-out option that instructs recipients to call us if they are not interested and we will remove their name from our list. The letter will also explain that if we do not receive an opt-out call from them within a week, we will call them to explain the study further and invite them to participate. To facilitate recruitment, we plan to inform potential candidates that:

- Each participant will receive the OLP at some point during the 7-week study.
- Free parking will be available to help alleviate the associated costs of participation.
- Each participant completing up 4 study visits will receive up to $75.00 in the form of cash in appreciation for their time and effort.

**Screening**: During a telephone screening, prospective participants will be asked to rate their current fatigue severity (0=no fatigue; 10=extremely severe fatigue). Those who report moderate fatigue (≥4) and who meet the other inclusion criteria (see Section 3.4) will be told that they qualify for the study after information is verified in IMPACT. The participants will be informed that:

- They will receive “placebo pills” at some point during the study.
- In several studies, placebos have been found to be just as effective, if not more so, than many drugs being used to relieve pain, to treat depression, anxiety, sleeplessness and fatigue when used in placebo-controlled clinical trials.
- Current recommended CRF treatments have not demonstrated adequate effect for relieving CRF symptoms.
- Placebos have been tested and found to be effective for relieving fatigue and pain in patients with symptoms related to IBS and migraines.
- They will be reimbursed for parking expenses.
- They will receive up to $75.00 in the form of cash for participating in the study.

**Enrollment:** After we identify eligible participants and receive a positive indication of willingness to participate, we will ask them for demographic information during the screening call. As each participant is verified and enrolled into the study, he or she will be scheduled for a total of 4 clinical site visits. Once the participant is enrolled, we will mail all relevant measurement instruments, instructions and consent forms prior to the first scheduled clinical site visit or Day 1 of the study.

**3.3 Informed Consent**

Upon enrollment and before Day 1 of the study, we will mail the Informed Consent form to the enrolled participant to allow adequate time for review and question formulation. On Day 1 of the study at the beginning of the study visit, the research specialist will review the Informed Consent form with the participant and address all questions and concerns. If the participant still chooses to participate in the study, we will acquire his or her witnessed signature. We plan to assure that the Inform Consent explains the participants’ rights, in clear, simple language, that includes:

- Information on the type of study this is and why we are doing it.
- Information on the procedures we will use, as well as a description of the placebo pill, instructions on how to take the prescribed placebos and how to use the saliva collection kit.
- Information on any known discomforts or risks, any associated costs (i.e., transportation) and any associated benefits.
- Any standards of care that might provide relief even if the prospective participant chooses not to participate in the study, as well as how the risks and benefits of such options compare to placebos.
- Any clinically effective treatment for CRF that becomes available during this study.
- The opportunity to ask any questions about the study or the procedures involved.
- The opportunity to be told of new findings that could change willingness to consent.
- The participant’s right to withdraw consent to participate at any time, without penalty or retribution.
- Protections for and rights of participants provided by GINA regarding the collection and analysis of DNA specimens.
- What kind of specimens will be collected and the means of collection.
- What type of research will be done with the specimens.
- Whether the biological specimens will be shared with other investigators,
- Whether biological specimens will be coded or anonymized (no way of tracing back to participant/uncoded or code destroyed).
- Whether the participant may be contacted for additional consent.
- How long, if known, the biological specimens will be stored. (Short-term: current protocol only or other current research; Long-term: future studies on disease or condition, repository, etc.).
- Foreseeable risks or benefits to participants in the collection, storage and subsequent research use of specimens.
- What will be done with the biological specimens if the participant refuses permission (“anonymized”—stripped of identifiers--or destroyed?).
- What will be done with the research results. (Research results should not be placed in the individual participant’s medical record.)
- The right to receive a copy of any consent form used in relation to the study.
- The time and opportunity to decide freely whether to consent or not consent to participate in the study.

**3.4 Inclusion, Exclusion, and Withdrawal Criteria**

**Inclusion criteria**:

- CSs who are at least 19 years of age with Stage II-IV cancer who have completed primary treatment ≥ 6months and within 10 years and have no evidence of disease;
- Report ≥4 (moderate fatigue) on a 0-10 fatigue severity rating scale;
- Agree not to change any of their medications or treatments during the study. (Should medication change be medically necessary, we will note this and incorporate it into the data analysis. However, given the short duration of the study, we do not anticipate medication changes);
- Willingness to make 4 clinical site visits over the course of the 49-day study.

**Exclusion criteria**:

- Stage I disease;
- Anemia of Hg<8gm/dl;
- Uncontrolled diabetes, COPD, hypertension, ischemic heart disease, liver/kidney disease, autoimmune disease or psychiatric or cognitive disorders.

**Withdrawal criteria:**

This is an investigator-initiated, uncomplicated, randomized crossover trial, and the risks/hazards are considered minimal. If a study participant desires to withdraw from the study, for whatever reason, they will be withdrawn.

**3.5 Randomization and Interventions (if applicable)**

A randomized assignment will be placed in an envelope that will be opened by the intervention administrator during the participants’ first clinical site visit (Day 1 of the study). Until that time, the intervention administrator will be blinded to the randomized assignment until it is revealed to the participant. However, because of the “open disclosure” of placebo use, study assignments cannot be blinded to the intervention administrator or to the participant once the envelope is opened. To control bias, the intervention administrator will have limited contact with the participants after Day 1 with the exception of a brief “re-orientation” on Day 28 at the beginning of Period 2 .

On Day 28, participants originally assigned to Group_2_ will receive the OLP and be prescribed two placebo pills twice a day to take over the course of 3 weeks (21 days). The participants who were randomly assigned to Group_1_ will serve as the NO OLP observational controls over the course of 3 weeks (21 days).

Figure 2: Crossover Study Arms

**Intervention: Placebo Orientation Meeting (Day 1 of Study / 1^st^ Clinical Site Visit)**

At the beginning of the first clinical site visit (Day 1 of Study) the research specialist will acquire informed consent from the participant. Once the participant has provided his or her consent, the research specialist will review the four questionnaires with the participant, address any questions or concerns and ask the participant to complete each instrument. After reviewing the questionnaires for missing data, the research specialist will label each completed questionnaire with the appropriate study numeric identifiers and store the questionnaires in a secured unit for future analysis. The research specialist will then instruct the participant on how to use the ORAGENE® Discover (OMR-500) saliva collection test kit and a saliva sample will be collected, labeled with the appropriate study identifiers and stored in a secured unit for future analysis. The research specialist will schedule all 3 subsequent clinical site visits (Days 21, 29 and 49) and introduce the participant to the intervention administrator.

The intervention administrator will deliver a persuasive rationale for the OLP to the participant as part of the OLP Orientation on Day 1. The persuasive rationale (OLP orientation) is a critical component of this study and will be presented to participants in a way that fosters positive expectations, without deception, when a placebo is delivered. The script for the persuasive rationale includes dialogue that:

- Supports positive expectations and trust with an accurate description of what is known about placebo effects;
- Allows for contradictory thoughts including doubt and disbelief; and,
- Encourages adherence to the medical ritual of pill taking.

Four main discussion points will be included in *a priori* script that takes approximately 20 minutes to deliver (see Table1). The introduction will begin with a question:

- “Have you ever heard about placebo effect prior to our discussion on the phone?” followed by a natural give-and-take discussion.
- The practitioner will explain (or support the patient’s understanding) that “placebo pills are made of an inert substance, like sugar or starch, that has been shown in clinical studies to produce significant improvement in a variety of symptoms including fatigue and pain, through mind-body, self-healing processes that our bodies can naturally do.”
- This exchange will lead the intervention administrator to explain the four main discussion points of the persuasive rational.

| **Table 1: Four Discussion Points of the Placebo Orientation Meeting** |
| --- |
| **Placebo Effect/Expectancy Building**: Explain how Medicine is learning to respect the power of placebo effect and how our team has demonstrated that placebo treatment can lead to substantial relief whether placebo is concealed or unconcealed. **This segment is designed to build patient expectancies.** |
| **Conditioning**: Explain how the body can respond automatically to placebo pills naturally by releasing chemicals like dopamine to relieve pain. Placebos have been shown to produce physiological responses that are independent of conscious belief. We will mention examples of conditioned placebo responses such as immunology and endocrinology as well as pain conditions like those associated with migraine headaches, arthritis and irritable bowel syndrome. **This segment seeks to reassure patients that response to placebo can produce legitimate changes in physiology.** |
| **Realistic Attitude Towards Belief/Disbelief:** Explain that positive expectations can be helpful but not necessary. Participants need to be comfortable with contradiction and ambiguity because their beliefs may way and wane during the trial. **This segment seeks to reassure patients that belief and disbelief are both compatible with placebo response and comfort with either is important.** |
| **Emphasize Automatic Importance of Adherence**: Explain that studies show that people who take placebo pills faithfully do much better than those who do not. **Our experience and pilot work suggests that enlisting patient commitment to performing the ritual of the treatment may be more important than the cognitive components of the script. Adherence also supports the conditioning dimension of the placebo.** |

The delivery of the persuasive rationale will be audio-recorded in all patients for use in establishing fidelity to treatment. Because of the importance of the interaction between the intervention administrator and the participant, all interactions will take place in the context of a warm, supportive and natural interaction. The importance of the no-treatment control group will also be discussed and questions will be answered. After the placebo orientation, the envelope with the randomized assignment will be opened and both the participant and the intervention administrator will learn which group the participant will join.

**Group_1_**: Participants assigned to Group_1_ will be given a pre-filled, twice-per-day weekly pill organizer. The intervention administrator will instruct Group_1_ to “take 2 pills in the morning with breakfast and two pills in the evening before bedtime.” The placebo pills will be blue and maroon gelatin capsules filled with non-absorbable Avicel® (Microcrystalline cellulose), a common inert excipient for pharmaceuticals. Prior to departing, the participant will ingest two of the placebo pills and be given a medication diary with instructions to record each time placebos are taken so that a pill count can be made.

**Group_2_**: Participants assigned to Group_2_ will be reassured that they will receive the OLP during Period 2 of the study. The intervention administrator will also reiterate the importance of the role of observational controls.

Parking tickets will be stamped and participants will be provided contact information for study team member and IRB should they have questions or concerns. They will be given a reminder card with their next appointments (Day 21, Day 29 and Day 49). They will also be told to expect a follow up telephone call from a study team member midway through Period 1 on or about Day 11. The estimated time for the first clinical site visit (Day 1) is 60 minutes.

**Mid-Point Check-In Call:** participant is called to check on condition. Calls lasts < 5 minutes.

**End of Period 1 (Day 21 of Study / 2^nd^ Clinical Site Visit**): Study participants will be instructed to return to the clinical site on Day 21 of the study. At that time, the research specialist will collect the medication diaries and address any questions or concerns. The research specialist will administer the 4 questionnaires that will be labeled with the appropriate study identifiers and stored in a secured unit for future analysis. Participants will be reminded of their next clinical site visits (Day 29 and Day 49) and parking tickets will be stamped. Once again, participants will be reminded of the value of their participation. The estimated time for the 2^nd^ clinical site visit is 25 minutes.

**Beginning of Period 2 (Day 29 of the Study / 3^rd^ Clinical Site Visit):** On Day 29, participants will return to the study site to begin Period 2 of the trial. The research specialist will review the 4 questionnaires with the participant, address any questions or concerns and ask the participant to complete each instrument. The research specialist will label each completed questionnaire with the appropriate study numeric identifiers and store the questionnaires in a secured unit for future analysis. The intervention administrator will meet with participants to explain procedures for Period 2.

**Group_1_** : participants will be informed that they are now the OC and the value of that role will be reiterated. They will be reminded of their next clinical site visit and told to expect a follow up call at some point (on or about Day 39). Parking tickets will be stamped and the participant will be provided a reminder of the next (and final) clinical site visit (Day 49 of the study).

**Group_2_** : participants will be informed that they will now receive the OLP. The intervention administrator will re-present a brief version of the rationale for the OLP and remind participants of the 4 main points from the original orientation meeting. Participants will be encouraged to ask questions and discuss concerns. Participants assigned to Group_2_ will be given a pre-filled, twice-per-day weekly pill organizer. The intervention administrator will instruct Group_2_ to “take 2 pills in the morning with breakfast and two pills in the evening before bedtime.” The placebo pills will be blue and maroon gelatin capsules filled with non-absorbable Avicel (Microcrystalline cellulose), a common inert excipient for pharmaceuticals. Prior to departing, the participant will ingest two of the placebo pills and be given a diary with instructions to record each time placebos are taken so that a pill count can be made. They told to expect a follow up call at some point (on or about Day 39). Parking tickets will be stamped and the participant will be provided a reminder of the next (final) clinical site visit (Day 49).The estimated time for the Day 29 clinical site visit is 35 minutes.

**Mid-Point Check-In Call:** participant is called to check on condition. Calls lasts < 5 minutes.

**End of Period 2/End of Study (Day 49):** Study participants will return to the clinical site on Day 49 of the study. At that time, the research specialist will collect the medication diaries and address any questions or concerns. The research specialist will administer the remaining questionnaires and assure that all questionnaires are stored in a secured unit for future analysis. The intervention administrator will meet briefly with participants to thank them for their participation and to distribute cash incentives. Parking tickets will be stamped. The estimated time for this visit is 25 minutes.

**3.6 Measures**

To qualify for the study, CSs must have a self-assessed fatigue severity level of >4 on a 0-10 scale with 10 being “extremely severe fatigue”. In addition to demographics, 3-questions will be asked during the screening process that will help assess feasibility and acceptability. OLP treatment effect will be measured with 4 questionnaires administered to participants Day 1 (Baseline) Day 21, Day 28 and Day 49 (End of Study). We will also determine the presence of the COMTVal18Met genotype.

**3.6.1 Primary Study Aim Outcomes**

**Specific Aim 1** evaluates the feasibility of recruiting and enrolling CSs into an OLP and the acceptability by CSs of an OLP for the treatment of CRF. We propose to measure these two outcomes as follows:

- Feasibility: among eligible CSs who inquire about our study, we will calculate the proportion of those who, after completing the screening procedure, (1) sign the consent form, (2) decline to participate and (3) participate but drop out. We will gather qualitative information from CSs concerning their reasons for declining to participate or dropping out. We will also investigate whether the screening questions pertaining to outcome expectancy and credibility associate with study retention and/or placebo responses, as well as whether randomized assignment associates with retention.

During the screening process, prospective enrollees will be asked 3 questions that pertain to credibility belief, outcome expectancies, and acceptability of an OLP. The rationale for asking these questions is to assess the feasibility of an OLP study for CSs, and to assess whether the responses to credibility beliefs, outcome expectancy predict acceptability and subsequent placebo responses (which may have implications for selecting patients for future clinical trials).

- **Credibility Belief**: “Based on what I have said today, how logical does taking placebo pills to make help someone feel better seem to you?” Responses will range from 0 (makes no sense at all) to 10 (makes perfect sense).
- **Outcome Expectancy**: “At this point, how successful do you think this treatment will be in reducing your fatigue?” Responses are measured on a 0% to 100% scale.
- **Acceptability**: “Based on my description of the study, are you still interested in participating?” Responses will be “yes” or “no”, or “not sure”. Those who respond “not sure” will be given 48-hours to decide if they wish to participate and will receive a follow-up call.
- Acceptability will also be measured by
- Accrual Rate
- Retention Rate
- Placebo Pill Adherence Rate

**3.6.2 Secondary Study Aim #1 Outcomes**

**Specific Aim 2** tests the hypothesis that an OLP reduces symptoms severity of CRF and improves perceived wellbeing and quality of life for CSs compared to an observational control. To assess effect, we will administer the following measures to measure specific outcomes related to CRF:

- **FACT-F** is a 41-item questionnaire that assesses quality of life concerns pertinent to cancer patients. It has evidenced good internal consistency reliability and concurrent validity that measures the following domains:

Physical well-being (7 items)

Social/Family well-being (7 items)

Emotional well-being (6 items)

Functional well-being (7 items)

Fatigue (13 items)

- **Multidimensional Fatigue Symptom Inventory** (MFSI-SF 30) is an instrument that considers the multidimensional conceptualization of cancer-related fatigue beyond symptom severity and intensity. In terms of validity, correlations between the 5 subscales of the MFSI-SF (listed below) and 2 measures of fatigue have demonstrated excellent concurrent validity and correlations with a measure of physical well-being provided support for convergent validity.

Global Symptom Severity

Physical Health

Emotional Health

Mental Health

- **Fatigue Symptom Inventory (FSI)** assesses the frequency and severity of fatigue as well as its perceived disruptiveness. Frequency is measured as the number of days in the past week (0–7) respondents felt fatigued as well as the percentage of each day on average they felt fatigued (0 = none, 10 = entire day). Perceived severity is measured on 4 separate 11-point scales (0 = not at all fatigued, 10 = as fatigued as I could be) that assess most, least, and average. Perceived disruptiveness is measured on 7 separate 11-point scales (0 = no interference, 10 = extreme interference) that assess the degree to which fatigue in the past week was judged to interfere with general level of activity, ability to concentrate, relations with others, enjoyment of life, and mood. Interference ratings are also summed to yield a total disruptiveness score**.**
- **Medical Outcomes Study) (MOS SF-36**) is a set of generic, coherent, and easily administered quality-of-life measures. These measures are widely utilized by managed care organizations and by Medicare for routine monitoring and assessment of care outcomes in adult patients.

The estimated completion time for the questionnaires outlined above is 25 minutes.

**3.6.3 Supplementary Aim Outcomes**

**This supplementary aim** is intended to evaluate variances of COMTVal18Met genotype that may serve as potential biomarkers for placebo responders. Should significant OLP effects be obtained, we will evaluate whether variants of COMT Val18Met (met/met, met/val and val/val alleles) associate with placebo responsiveness.

**3.6.4 Primary Endpoint**

To evaluate the feasibility of recruiting and enrolling CSs into an OLP and the acceptability of CSs of an OLP for the treatment of CRF.

**3.6.5 Covariates**

We will analyze the following covariates to determine effects on outcomes:

- Age
- Sex
- Race
- Time Since Last Treatment

**3.7 Statistical Methods**

The statistical analysis will be conducted under the direction of Dr. Tapan Mehta. This pilot study will provide us important information concerning the acceptability and feasibility of OLP for treating CRF. It will also provide information on study design, retention and outcomes assessment.

**Power Calculation**: Our sample size calculations were based on minimal assumptions. Hence for each outcome, our planned sample size calculations indicated that we would have power of 80% assuming a two-tailed two-sample t-test on the outcomes at day 21, Type 1 error rate of 0.05, we would need a sample size of 80 to detect an effect size of 0.64. Techniques such as ANCOVA or two–sample independent t-test comparing change scores would be powerful or require less sample size to detect the same effect size depending on the correlation between baseline and follow-up measures

**Statistical Analysis**

**Specific Aim 1:** We will evaluate the feasibility of recruiting and enrolling CSs into an OLP intervention.

Feasibility: among eligible CSs who inquire about our study, we will calculate the proportion of those who, after completing the screening procedure,

- sign the consent form;
- decline to participate;
- participate but drop out.

During the screening process, prospective enrollees will be asked 3 questions that pertain to credibility belief, outcome expectancies, and acceptability of an OLP. The rationale for asking these questions is to assess the feasibility of an OLP study for CSs, and whether credibility beliefs, outcome expectancy predict acceptability (which may have implications for selecting patients for future clinical trials). We will use an analysis of variance (ANOVA) to determine if credibility belief and/or outcome expectancy predicts acceptability.

- **Credibility Belief**: “Based on what I have said today, how logical does taking placebo pills to make help someone feel better seem to you?” Responses will range from 0 (makes no sense at all) to 10 (makes perfect sense).
- **Outcome Expectancy**: “At this point, how helpful do you think this treatment will be in reducing your fatigue?” Responses are measured on a 0% to 100% scale.
- **Acceptability**: “Based on my description of the study, are you still interested in participating?” Responses will be “yes” or “no”, or “not sure”. Those who respond “not sure” will be given 48-hours to decide if they wish to participate and will receive a follow-up call.

Acceptability: This will be determined proportionally by:

- **Accrual Rate**: baseline = 80 eligible participants
- **Retention Rate**: baseline = >75%
- **Adherence Rate**: placebo adherence by actual pill count and medication diary entries compared to an anticipated baseline rate of 100%.

For the analysis of acceptability, we will use a chi-square test, or Fisher’s exact test when the assumptions for the chi-square are not tenable. We will also apply log linear models if necessary to adjust for covariates.

**Specific Aim 2:** We will test the hypothesis that an OLP intervention reduces symptoms severity of CRF and improves quality of life for CSs compared to an observational control. The primary analysis for this aim will consist of mixed models repeated measures analysis, such as repeated measures analysis of covariance. This will allow the investigators to properly test for the absence of a carryover effect as well as account for the fact that all patients will receive both treatments (i.e., this type of analysis allows each patient to serve as his/her own control).

**Supplementary Aim:** evaluates the presence or absence of the COMTVal18Met genotype as a correlate for improved fatigue conditions and quality of life. We will measure this using a logistic regression model to determine associations. In addition, we will calculate the odds ratio and corresponding 95% confidence intervals.

**Multiple Comparisons Tests (Post Hoc Test):** To account for multiple comparisons, we will apply a Bonferroni procedure and control the family-wise error rate at .05.

**Missing Data**: Data deemed missing at random (MAR) will be handled using multiple imputation techniques with the Bayesian based Markov-Chain Monte-Carlo (MCMC) method. In supplementary analysis, we will use independent t-tests and chi-square to compare completers versus dropouts.

**4.0 DATA AND SAFETY MONITORING**

Kevin Fontaine and Theresa Hoenemeyer will be responsible for reviewing and monitoring all study data and any adverse events. Dr. Posey will be consulted as needed. Theresa Hoenemeyer will meet with any study personnel weekly to evaluate study progress, including periodic assessments of data quality and timeliness, participant recruitment, accrual and retention, participant risk versus benefit, and other factors that can affect study outcomes. Unanticipated problems and adverse events will be reported to the UAB IRB according to policies.

All data related to this study will be password protected and securely stored at UAB’s Comprehensive Cancer Center. Related database will not contain any personal identifying information to link to the subject. The specimens will have a unique identifying code (study ID number). Clinical information will be stored in a separate and password-protected database that links samples and other information to the subjects. The specimens and related data spreadsheets will contain only the subject ID codes to prevent the ability to link to personal identifiers. The database requires a secure password and access to this password will be limited. Access to these files will be limited to study team members and only the research staff members who have received IRB approval are allowed to access the database.

**Potential Risks**: The risks of this study are judged to be minimal and the risk/benefit ratio to be favorable. Subjects may experience normal fluctuations in their disease/treatment long-term effects (i.e., fatigue, pain). Since we are not administering any active ingredients and the subjects will be evaluated 6 times over a 49-day period, there is minimal risk to the subject. Study participants may discontinue participation in all or some of the study activities at any time if he/she feels uncomfortable or wishes to do so.

We have study personnel with experience in studies of CSs. Theresa Hoenemeyer, the principal investigator, and Dr. Fontaine will assure the safety of study participants. We will monitor the safety of subjects at each visit for the following: (1) adverse effects, (2) symptom evaluation, and (3) concomitant medication use. Should it be determined that continued participation in this study is contra-indicated for a participant, we will withdraw the participant from the study. The participant will be advised to contact his or her physician’s office to report symptoms that are not consistent with cancer-related fatigue. Given that this is a non-invasive study of short duration (49 days), we do not anticipate withdrawals to be common.

**4.1 Ethical Considerations**

We will conduct this study in accordance with Good Clinical Practice, the Declaration of Helsinki, and 21CFRPart50 – Protection of Human Patients, Part 56 – Institutional Review Boards, and the other applicable local ethical and legal requirements. The Ethics Review Committee/Institutional Review Board (IRB) will be constituted according to Code of Federal Regulations (CFR). Written unconditional approval will be obtained by the UAB IRB before commencement of the study. The IRB will be informed by the Principal Investigator of all subsequent protocol amendments and of serious or unexpected adverse events occurring during the study that are likely to affect the safety of the patients or the conduct of the study. Approval for such changes will be transmitted in writing to the Sponsor (UAB) via the Principal Investigator. The Principal Investigator or designee will notify the IRBs and investigators when the study is placed on “hold”, completed, or closed to further patient enrollment.

**4.2 Confidentiality**

Every effort will be made to maintain the confidentiality of each participant’s medical record and information attained by the investigator and the IRB to the extent permitted by law. Only group information without personal identifiers will be included when submitting manuscripts for publication. Information about the participant will not be shared without their permission. All information gathered from this study will be treated as medical record with the same degree of confidentiality.

**5.0 REFERENCES**

1. Ryan J., Carroll J., Ryan E., et al. (2007). Mechanisms of cancer-related fatigue. The Oncologist, 12(Supplement 1):22-34. Retrieved on 02/10/15 from <http://www.ncbi.nlm.nih.gov/pubmed/17573453> [↑](#endnote-ref-2)
2. National Cancer Institute. (2014). Fatigue PDQ® General Information About Fatigue. Updated: March 14, 2014. Retrieved on 12/07/14 from <http://www.cancer.gov/cancertopics/pdq/supportivecare/fatigue/Patient/page1>. [↑](#endnote-ref-3)
3. Fosså S., Dahl A., Loge J. (2003). Fatigue, anxiety, and depression in long-term survivors of testicular cancer. J Clin Oncol 21 (7), 1249-54. DOI: 10.1200/JCO.2003.08.163. [↑](#endnote-ref-4)
4. Miaskowski C. & Portenoy R. (1998). Update on the assessment and management of cancer-related fatigue. Principles and Practice of Supportive Oncology Updates, 1(2), 1-10. [↑](#endnote-ref-5)
5. Irvine D., Vincent L., Bubela N., Thompson, L., Graydon, J. (1991). A critical appraisal of the research literature investigating fatigue in the individual with cancer. Cancer Nurs, 14 (4), 188-99. [↑](#endnote-ref-6)
6. Vogelzang N., Breitbart W., Cella D., Curt G., Groopman J., Horning S., Itri L., Johnson D., Scherr S., Portenoy R. (1997). Patient, caregiver, and oncologist perceptions of cancer-related fatigue: results of a tri-part assessment survey. The Fatigue Coalition. Sem in Hematol, 34 (3 Suppl 2), 4-12. [↑](#endnote-ref-7)
7. Detmar, S., Aaronson, N., Wever, L., Muller. M. & Schornagel, J. (2000). How are you feeling? Who wants to know? Patients' and oncologists' preferences for discussing health-related quality-of-life issues. J Clin Oncol, 18 (18), 3295-301. [↑](#endnote-ref-8)
8. Costantini. M, Mencaglia, E. , Giulio, P. , Cortesi, E., Roila, F., Ballatori, E., Tamburini, M., et al. (2000). Cancer patients as 'experts' in defining quality of life domains. A multicentre survey by the Italian Group for the Evaluation of Outcomes in Oncology (IGEO). Qual Life Res, 9 (2), 151-9. Retrieved from: [http://web.a.ebscohost.com/ehost/pdfviewer/pdfviewer?sid=0a4f035e-89ad-49de-b609- b54df9402283%40sessionmgr4005&vid=2&hid=4212](http://web.a.ebscohost.com/ehost/pdfviewer/pdfviewer?sid=0a4f035e-89ad-49de-b609-%09b54df9402283@sessionmgr4005&vid=2&hid=4212) [↑](#endnote-ref-9)
9. Curt G., Breitbart W., Cella D., Groopman J,, Horning S,, Itri L., Johnson D., Miaskowski C., Scherr S., Portenoy R., Vogelzang N. (2000). Impact of cancer-related fatigue on the lives of patients: New findings from the Fatigue Coalition. The Oncologist, 5 (5) 353–360. Retrieved from: <http://www.ncbi.nlm.nih.gov/pubmed/11040270> [↑](#endnote-ref-10)
10. Kaptchuk, T., Friedlander, E., Kelley, J., Norma, M., Singer, J., Kowalczykowski, M. Miller, F., Kirsch, I., Lembo, A. (2010). Placebos without Deception: A Randomized Controlled Trial in Irritable Bowel Syndrome. PLOS, 5(12). DOI:10.1371/journal.pone.0015591. [↑](#endnote-ref-11)
11. Kelley J., Kaptchuk T., Cusin C., Lipkin S., Fava M. (2012). Open-label placebo for Major Depressive Disorder: a pilot ramdomized controlled trial. Psychother Psychosom, 81(5), 312-314. DOI:10.1159/000337053. [↑](#endnote-ref-12)
12. Kam-Hansen, S., Jakubowski, M., Kelley, J., Kirsch, I., Hoaglin, D., Kaptchuk, T. & and Burstein, R. (2014). Altered Placebo and Drug Labeling Changes the Outcome of Episodic Migraine Attacks. Sci Transl Med, 6 (218), 1-7. [↑](#endnote-ref-13)
13. Hall, K. T., & Kaptchuk, T. J. (2013). Genetic biomarkers of placebo response: what could it mean for future trial design? Clinical Investigation, 3(4), 311–314. doi:10.4155/cli.13.8. [↑](#endnote-ref-14)
14. Bower, J. E., & Lamkin, D. M. (2013). Inflammation and cancer-related fatigue: Mechanisms, contributing factors, and treatment implications. *Brain, Behavior, and Immunity*, *30*(0), S48–S57. doi:10.1016/j.bbi.2012.06.011. [↑](#endnote-ref-15)
